# Probing milk extracellular vesicles for intestinal delivery of RNA therapies

**DOI:** 10.1186/s12951-023-02173-x

**Published:** 2023-11-03

**Authors:** Yunyue Zhang, Mona Belaid, Xiang Luo, Armond Daci, Rinë Limani, Julia Mantaj, Matthias Zilbauer, Komal Nayak, Driton Vllasaliu

**Affiliations:** 1 Institute of Pharmaceutical Science, School of Cancer and Pharmaceutical Science, King’s College London, London, SE1 9NH UK; 2 Faculty of Medicine, University of Prishtina “Hasan Prishtina”, 10000 Prishtina, Kosovo; 3 Present Address: School of Life Sciences, Faculty of Science and Engineering, Anglia Ruskin University, Cambridge, CB1 1PT UK; 4 Wellcome-MRC Cambridge Stem Cell Institute, University of Cambridge, Cambridge, CB2 0AW UK; 5 Department of Paediatrics, University of Cambridge, Cambridge, CB2 0QQ UK

**Keywords:** Extracellular vesicles, Oral nucleic acid delivery, siRNA, Inflammatory bowel disease, Intestinal epithelium

## Abstract

**Background:**

Oral delivery remains unattainable for nucleic acid therapies. Many nanoparticle-based drug delivery systems have been investigated for this, but most suffer from poor gut stability, poor mucus diffusion and/or inefficient epithelial uptake. Extracellular vesicles from bovine milk (mEVs) possess desirable characteristics for oral delivery of nucleic acid therapies since they both survive digestion and traverse the intestinal mucosa.

**Results:**

Using novel tools, we comprehensively examine the intestinal delivery of mEVs, probing whether they could be used as, or inform the design of, nanoparticles for oral nucleic acid therapies. We show that mEVs efficiently translocate across the Caco-2 intestinal model, which is not compromised by treatment with simulated intestinal fluids. For the first time, we also demonstrate transport of mEVs in novel 3D ‘apical-out’ and monolayer-based human intestinal epithelial organoids (IEOs). Importantly, mEVs loaded with small interfering RNA (siRNA) induced (glyceraldehyde 3-phosphate dehydrogenase, GAPDH) gene silencing in macrophages. Using inflammatory bowel disease (IBD) as an example application, we show that administration of anti-tumour necrosis factor alpha (TNFα) siRNA-loaded mEVs reduced inflammation in a IBD rat model.

**Conclusions:**

Together, this work demonstrates that mEVs could either act as natural and safe systems for oral delivery or nucleic acid therapies, or inform the design of synthetic systems for such application.

**Graphical Abstract:**

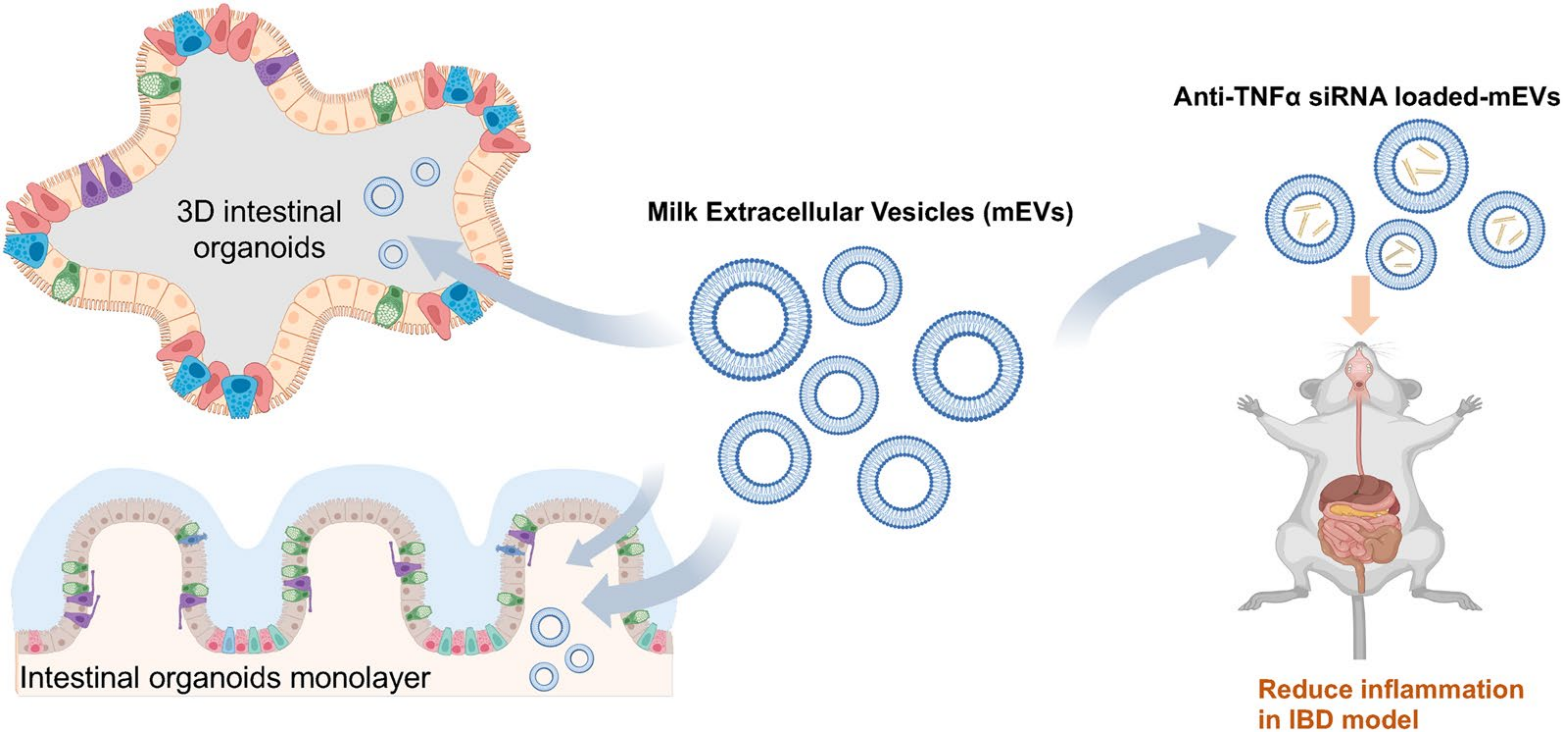

**Supplementary Information:**

The online version contains supplementary material available at 10.1186/s12951-023-02173-x.

## Background

Ingestion is the most desirable drug administration route. In addition to offering significant benefits in terms of access to medicines and patient convenience, oral administration is also amenable to effective local drug delivery in the gastrointestinal tract (GIT) as part of therapeutic management of GIT diseases, such as inflammatory bowel disease (IBD). Nanomedicine-based approaches have been investigated for oral delivery of nucleic acid therapies in intestinal inflammation [1–3], but most synthetic nanoparticles tend to suffer from poor stability in, and inefficient penetration across, the challenging biochemical and physical barriers of the GIT [4].

All cells export part of their proteins, lipids, and nucleic acids into the extracellular space *via* the release of various types of extracellular vesicles (EVs), which play a crucial role in intercellular communication. As part of their role of cargo transfer between cells, EVs are highly capable of crossing biological barriers. Because of this property, EVs, and particularly exosomes (a subtype of EVs), have attracted significant interest as potential drug delivery systems [5]. EVs isolated from bovine milk (mEVs) are potentially highly interesting systems for delivery of drugs with poor oral bioavailability, such as biologics. Based on observations that mEVs exert bioactivity, both locally in the GIT and systemically, it is thought that they both survive the hostile conditions in the GIT [6, 7] and translocate across the human intestinal mucosa [8–10].

Interestingly, mEVs per se have been investigated for their therapeutic potential in IBD and were found to ameliorate colitis in a murine model of the disease [11]. This therapeutic effect of mEVs is thought to arise from beneficial micro RNA (miRNA) and cytokine cargo, such as miRNA-148 [12] and transforming growth factor beta (TGF-β, an immune-suppressive cytokine) [13]. While this early indication of therapeutic potential of native mEVs needs to be confirmed in more human-relevant animal models (and ultimately humans), here we used innovative in vitro approaches, including a state-of-the-art model of the human intestinal epithelium, to examine the utility of mEVs as potential vehicles, or models of nanoparticles, for oral delivery of nucleic acid therapies.

We initially examined the interaction of mEVs with the key drug delivery barriers in the intestine, namely the intestinal biofluid and epithelium, followed by probing their potential for oral delivery of biologics in IBD (using siRNA as exemplar therapeutic). mEVs were isolated and characterized, including in the presence of simulated intestinal fluids. Intestinal cell uptake and transport of mEVs was then confirmed in Caco-2 cells as an established in vitro intestinal model, together with assessing the effect of simulated intestinal fluid on their epithelial transport. Importantly, using novel intestinal epithelial organoid (IEOs) models of the human intestinal epithelium, we demonstrate epithelial translocation of mEVs in this highly biorelevant intestinal epithelial model. We show that mEVs efficiently transport across Caco-2 cells, which was not affected by their treatment with simulated intestinal fluids. We further demonstrate apical-to-basolateral translocation of mEVs in two separate human tissue derived IEO models, which were, unconventionally, cultured with an exposed apical surface. When loaded with a model siRNA molecule (anti glyceraldehyde-3-phosphate dehydrogenase, GAPDH) by electroporation, mEVs induced gene silencing in a macrophage cell line, which was employed here given the central role of these immune system cells in mediating inflammation in IBD. Finally, a proof-of-concept in vivo study utilising anti-TNFα siRNA loaded mEVs demonstrated their ability to reduce inflammation in a rat model of IBD. This work therefore clearly highlights that mEVs could serve as highly effective carriers or inform the design of bio-inspired synthetic systems for oral delivery of nucleic acid therapies.

## Results and discussion

### mEV isolation and characterization

mEVs were initially characterized for size (Dynamic Light Scattering (DLS) and Nanoparticle Tracking Analysis (NTA)) and surface charge. These parameters are summarized in Table 1. The size of isolated mEVs falls within the range of those reported previously [13]. Transmission electron microscopy (TEM) was used to image the morphology of mEVs. Additional file 1: Fig. S1A depicts the typical cup-shaped structure of mEVs under negative staining [14]. Determining the expression of mEV protein markers (*via* the Exo-Check™ Array kit), Additional file 1: Fig. S1B  shows that the general markers of EVs such as CD63, CD81, ICAM (Intercellular Adhesion Molecule-1) and TSG101 (Tumor Susceptibility Gene 101) were expressed in mEVs, while ALIX (ALG-2-interacting protein X) is not apparent (this protein exists in EVs from colostrum and not mature milk [15]). Epithelial cell adhesion molecule (EpCAM), which is often found in cancer-derived EVs, was also not apparent in mEVs.

**Table 1:** Physicochemical parameters of bovine milk extracellular vesicles

Parameter & measurement method	Value
Size [nm] (DLS)^(a)^	136.9 ± 1.144
PdI^(b)^ (DLS)	0.155 ± 0.018
Size [nm] (NTA)^(c)^	152.0 ± 12.2
Zeta-potential [mV]	− 9.64 ± 1.04

^(a)^ DLS: dynamic light scattering; ^(b)^ PdI: polydispersity index; ^(c)^ NTA: nanoparticle tracking analysis. Data presented as mean ± SD (n = 3)

### Transport of mEVs across Caco-2 monolayers

Initial studies of intestinal transport of mEVs were carried out in Caco-2 monolayers as a commonly employed intestinal epithelial model. Comparisons were made with liposomes of similar size (~ 100 nm), as well as the fluorescent dye alone which was used to label mEVs. Additional file 1: Fig. S2 shows that the extent of transport was notably higher for mEVs compared to liposomes (over an order of magnitude) or the dye alone.

### Effect of intestinal fluids on mEV membrane stability and intestinal transport

Prior to studying the intestinal epithelial transport of mEVs in advanced and highly realistic intestinal models, we determined their stability in intestinal fluids, *via* size, and surface charge measurements. We intentionally focused on intestinal rather than gastric fluids given that the most appropriate way in which mEVs or mEV-like synthetic delivery systems would be administered orally is *via* enteric-coated capsules so to ensure that membrane-associated proteins of mEVs are protected in the harsh environment of stomach biofluid.

**Fig. 1:**
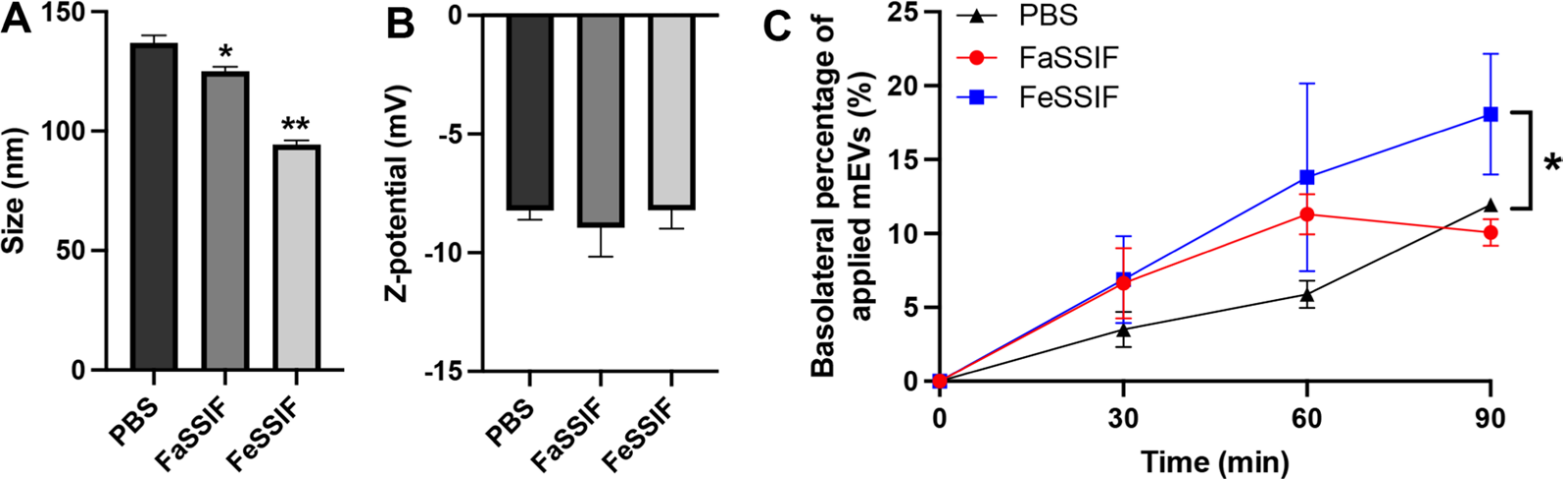
Effect of simulated intestinal fluids on bovine milk extracellular vesicles (mEVs). **A** size, **B** Z-potential, and **C** transport across differentiated intestinal epithelial Caco-2 monolayers. mEVs were treated with simulated intestinal fluids for 90 min. FaSSIF: Fasted State Simulated Intestinal Fluid; FeSSIF: Fed State Simulated Intestinal Fluid. Data shown as the mean ± SD, n = 3. * and ** indicate *p* < 0.05 and *p* < 0.01 compared with PBS group, respectively

There is evidence that cargo in mEVs (e.g. microRNAs) remains protected against degradation by low pH, RNases and treatment that mimics digestion in the GIT [6, 7]. However, studies reporting on the stability of mEVs and their content in the gut (which mainly come from the field of nutrition) tend to expose milk, rather than isolated EVs, to digestive conditions [6]. Figure 1A shows that while mEVs treated with fasted state simulated intestinal fluid (FaSSIF) displayed a similar size to those in PBS, fed state simulated intestinal fluid (FeSSIF) treatment resulted in a decreased diameter of mEVs. The surface charge (Z-potential) of mEVs was not compromised by simulated intestinal fluids (SIFs) digestion (Fig. 1B). This observation is similar to the findings reported by Kokkona et al. [16] on the effect of sodium cholate on the mean diameter of liposomes containing phosphatidylcholine and cholesterol, which decreased by approximately 20% in the presence of sodium cholate.

We next sought to understand whether the ability of mEVs to translocate across the intestinal epithelium is compromised upon treatment with SIFs. Figure 1C shows the transport of mEVs, post-treatment with SIFs, across polarised/differentiated Caco-2 monolayers. In all three groups, mEVs showed a remarkable ability to permeate across the Caco-2 monolayers. Interestingly, FeSSIF-treated mEVs possessed a slightly higher transport through the monolayers compared to FaSSIF-treated and untreated control. Specifically, approximately, 18% of FeSSIF-treated mEVs translocated across cell monolayers in 90 min, while FaSSIF-treated and untreated control showed a lower level of translocation, amounting to 10% of applied mEVs after 90 min. The calculated rate of mEV transport across intestinal epithelial monolayers was 12.0, 6.7 and 7.9% per hour for FeSSIF-treated, FaSSIF-treated and untreated mEVs, respectively, with the difference between FeSSIF-treated and untreated mEVs being statistically significant (*p* = 0.026). It is presently unclear why the transport rate of mEV across Caco-2 monolayers for FeSSIF-treated group was higher than that for FaSSIF-treated and control groups, although it may be related to the observed effect of FeSSIF on the size of mEVs.

It is noted that the SIFs employed in this work are simple models of the intestinal biofluid, which do not fully represent their composition. As such, they are derived of vital intraluminal components; for example, whilst native human intestinal fluid harbours a variety of bile salts, the SIFs used here comprise pure sodium taurocholate only [17]. These fluids additionally fail to replicate the intricate ultrastructure of postprandial human intestinal fluid (which includes mixed micelles and vesicles), likely due to the absence of lipids and lipolysis products. Furthermore, the intestinal movement and enzymatic degradation of food (which impact colloid formation) within the gastrointestinal tract are unaccounted for [18, 19]. Although this study supports previous evidence on the stability of mEVs in the GIT, ultimate evidence of the ability of mEVs to survive digestion should come from in vivo studies in the future.

### mEV transport across apical-out 3D IEOs

IEOs recapitulate the physiology, genetic signature and multicellular nature of the native intestinal epithelium [20]. The representation of all types of terminally differentiated intestinal epithelial cells, which serve important functions in barrier regulation, material absorption, mucus secretion, interaction with microbiota, gut-brain communication and host defense [21], is a key advantage of IEOs over cell line alternatives for modelling the intestinal epithelium. To evaluate the transport of mEVs across IEOs, we first cultured these using a typical culture condition, and using DAPT, we induced differentiation of IEOs so to promote the production of mucus (Additional file 1: Fig. S3) [22]. However, conventional culture of IEOs currently limits their application in the field of drug delivery since the apical/luminal side is not accessible by the user. To overcome this obstacle, recent studies have reported the culture of IEOs with exposed apical surface. To this end, a few studies have reported the culture of 3D apical-out human enteroids [22, 23]. However, to the best of our knowledge, IEOs have not previously been used to study the intestinal translocation of EVs, or indeed any other nanoparticles.

**Fig. 2:**
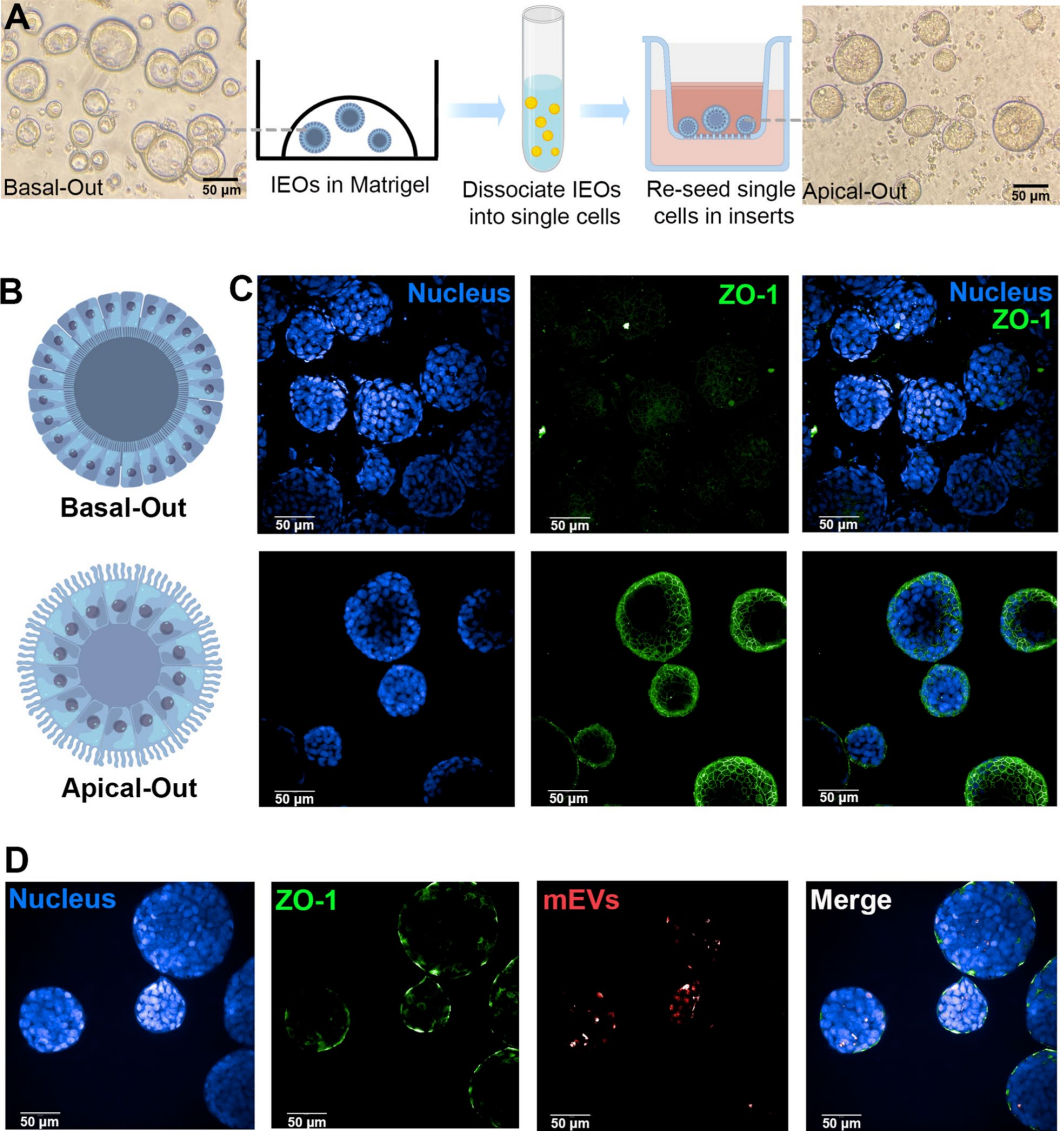
Apical-out culture of human (biopsy-derived) colon intestinal epithelial organoids (IEOs) and transport of milk extracellular vesicles (mEVs). **A** Brightfield image of IEOs cultured in Matrigel (basal-out) and PET transwells inserts (apical-out), and the schematic for development of apical-out IEOs from basal-out polarity. **B** Depicted schematic of basal-out and apical-out IEOs. **C** Confocal immunofluorescent staining images of IEOs with apical-out and basal-out polarity. **D** Confocal immunofluorescent staining images of mEVs transporting across apical-out IEOs after incubation for 4 h. Nucleus in blue (DAPI), apical zonula occludens (ZO-1) tight junction protein in green, and mEVs in red. Schematics were drawn by Figdraw

Based on a modified method reported by Co et al., we initially cultured 3D IEOs derived colon tissue with reversed polarity [22, 23]. The process of achieving successful in vitro culture of apical-out IEOs is shown in Fig. 2A. Confocal imaging revealed that when compared to normal basal-out IEOs, which have apical tight junction ZO-1 proteins expression in the interior of the cell clusters, in apical-out IEOs apical ZO-1 is distributed on the surface of IEOs clusters, facing outward (Fig. 2B, C), which confirmed the successful development of 3D apical-out IEOs model. We then applied fluorescently labelled mEVs to the apical-out IEOs model for four hours, followed by confocal imaging of uptake (Fig. 2D). In these systems, the fluorescence signal (red) of mEVs was clearly apparent within the interior of apical-out IEOs, which indicates transepithelial transport of mEVs from the exterior facing the apical side of 3D IEOs into their basolateral lumen.

### mEV transport across IEO monolayers

**Fig. 3:**
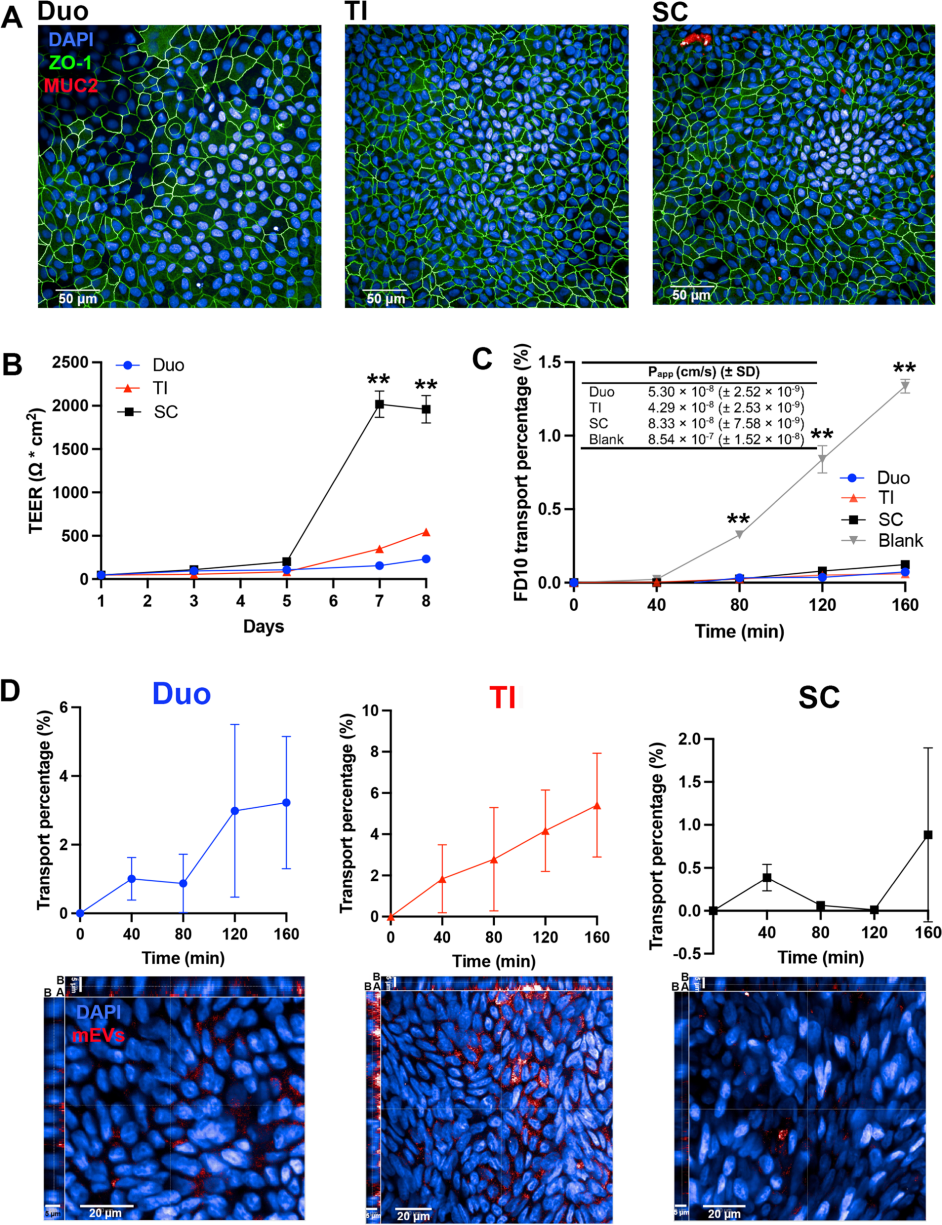
Culture of human intestinal epithelial organoids (IEOs) as 2D monolayers on Transwell inserts and epithelial transport of milk extracellular vesicle (mEVs). **A** Confocal immunofluorescent staining images of IEOs monolayers cultured on Transwell inserts for 8 days (differentiated at day 5), immunostained for the apical zonula occludens (ZO-1) tight junction protein (green), MUC2 mucin (red) and cell nucleus (DAPI, blue). **B** Transepithelial electrical resistance (TEER) of IEOs cultured as monolayers. **C** Transport percentage of fluorescein isothiocyanate–dextran with molecular weight of 10k (FD10) through IEOs monolayers and blank inserts with diluted basement membrane extract (BME2) coating, and insert table shows apparent permeability coefficient (P_app_) of FD10 through monolayers. **D** mEVs transport across IEOs monolayers including transport percentage shown on the upside and 3D confocal images on the bottom, where the apical side of the cells is marked by ‘A’ and the basolateral side ‘B’. Nuclei appear in blue and mEVs in red. IEOs were derived from biopsied tissue from different regions of human gastrointestinal system (‘Duo’: duodenum; ‘TI’: terminal ileum; and ‘SC’: sigmoid colon). Data shown as the mean ± SD, n = 3. ** indicates *p* < 0.01

In addition to demonstrating apical-to-basolateral transport of mEVs in 3D apical-out IEOs, we also determined their translocation in IEO monolayers originating from different gut segments (i.e. duodenum (Duo), terminal ileum (TI) and sigmoid colon (SC)). 2D monolayers enable easy access to the apical and basolateral sides and therefore the determination of transintestinal transport of material. To achieve this system, IEOs cultured in Matrigel were harvested for monolayer preparation at an optimized culture period, dissociated and single cells seeded on Transwell inserts. Additional file 1: Fig. S4 depicts the growth of IEO monolayers over a period of seven days, whereby the differentiation medium was added on day 5 and differentiated confluent IEOs monolayers developed by day 7. Figure 3A shows immunofluorescence staining images of Duo, TI and SC IEOs monolayers. ZO-1 fluorescence signal displays a characteristic ‘chicken-wire’ distribution of tight junctions. MUC2 signal is absent in Duo and TI monolayers, while it is weak in SC monolayers. The formation of electrically tight monolayers was also confirmed *via* TEER measurement. Figure 3B shows that the TEER values of Duo, TI and SC monolayers increased gradually with time during the first five days with culture in normal growth medium, while after the addition of differentiation medium on day 5, the TEER of monolayers from all three intestinal regions increased significantly, reaching beyond 200 Ω * cm^2^. To probe the barrier of IEO monolayers, we determined FD10 permeability in this system. Figure 3C shows a significantly lower permeability of FD10 across IEO monolayers compared with blank (no cell) inserts (coated with diluted BME2, similarly to cell-containing counterparts), and the apparent permeability coefficients (P_app_) of FD10 through IEO monolayers were lower than 10^−7^ cm/s, which confirms the barrier integrity of monolayers. Overall, the distribution of ZO-1 tight junction protein, high TEER and low permeability of FD10 together demonstrate the successful development of polarized Duo, TI and SC IEOs monolayers which are electrically tight and pose a barrier to transepithelial diffusion of a model macromolecule.

The data in Fig. 3D show mEVs accumulation on the basolateral side over time, reaching approximately 3% and 5% after 160 min across Duo and TI IEO monolayers, respectively (top). The cellular localization of mEVs following their incubation with IEO monolayers is shown on confocal images (Fig. 3D, bottom). The fluorescence signal associated with mEVs can be observed in the cell interior and across the vertical cross-sections of the monolayers, with more prominent distribution of fluorescence on the apical side. The accumulation of red mEVs fluorescence in TI monolayers was higher than Duo monolayers, which was consistent with the transport quantitation. As shown in Fig. 3D, SC, both the quantitative transport measurement and 3D confocal images show that mEVs transport across SC monolayers is the least efficient (compared to Duo and TI monolayers).

Data in Fig. 3 confirms the intestinal epithelium crossing characteristic of mEVs, also demonstrated in Caco-2 monolayers (Fig. 1) and ‘apical-out’ 3D IEOs (Fig. 2). Additionally and importantly, the data highlights that mEV transport across Duo- and TI-derived IEO monolayers is significantly higher than that in SC-derived monolayers. Although it is relatively well established that the intestinal barrier is more ‘leaky’ in the small intestine compared to the colon [24], attributed to differential expression of tight junctions [25], a markedly different transport profile of mEVs in SC compared to Duo and TI IEOs cannot be compared with the permeability of small molecular weight drugs, or indeed macromolecules. However, it has been reported that the permeability of actively transported compounds, d-glucose and l-leucine, is dramatically lower in the colon compared to the small intestine in a study utilizing human tissue in Ussing chambers [26]. This provides an interesting comparison, as although the mechanisms of intestinal epithelial transport of particulate mEVs are expected to be facilitated by different mechanisms to actively-transported molecules, it may be the case that the lower transport of mEVs in SC is linked to lower expression levels of the cellular machinery involved in their trafficking, although presently this is only a speculation and needs confirming in future studies.

### In Vitro transfection efficiency of siRNA-loaded mEVs

**Fig. 4:**
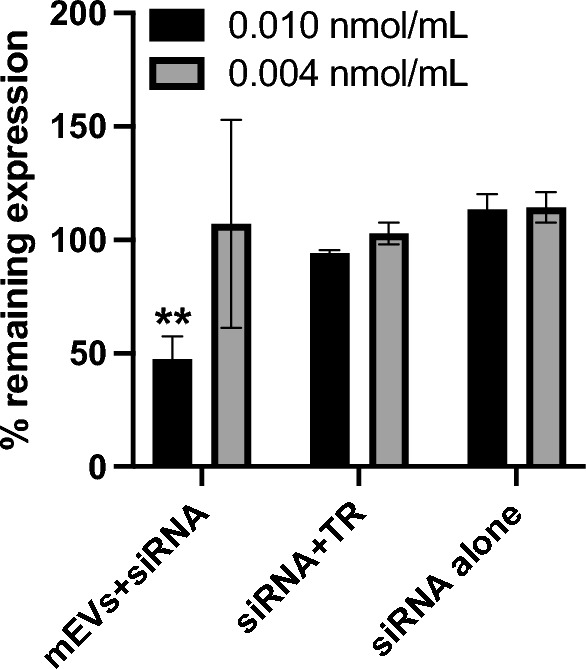
Expression levels of GAPDH in macrophages transfected with siRNA-loaded milk extracellular vesicles (mEVs) or siRNA with a commercial transfection reagent (TR), compared with siRNA alone. mEVs concentrations were 0.05 and 0.02 mg/mL corresponding to the siRNA concentration of 0.010 and 0.004 nmol/mL. Data are presented as mean ± SD (n = 3). ** indicates *p* < 0.01 compared with siRNA + TR and siRNA alone group

To establish whether mEVs could serve as potential systems for functional biotherapeutic delivery, we loaded siRNA into the vesicles *via* electroporation. The loading efficiency of siRNA into mEVs was calculated as 5.10 ± 0.55%. The gene silencing efficiency of siRNA loaded-mEVs was then evaluated on macrophages (J774A.1), given that macrophages in the lamina propria (i.e. under the epithelium) play a key role in inflammatory response in IBD [27] and therefore are a potential target of interest in IBD, achieved by a delivery system that permeates the intestinal barrier, such as mEVs. A model ‘housekeeping’ protein, GAPDH, was selected as a target for knock-down as it represents a commonly chosen target for these studies, and the GAPDH activity was measured by KDalert™ GAPDH Assay Kit to determine gene silencing [28, 29]. The gene silencing efficiency of GAPDH siRNA-loaded mEVs in macrophages is shown in Fig. 4. 0.05 mg/mL GAPDH siRNA loaded-mEVs (corresponding to 0.010 nmol/mL loaded siRNA) possessed around 50% silencing efficiency (48 h post-transfection), which was significantly higher than siRNA transfected with a commercial transfection reagent (~ 5% silencing efficiency) and negative control (siRNA alone).

### Efficacy of siRNA-loaded mEVs in a rat model of IBD

**Fig. 5:**
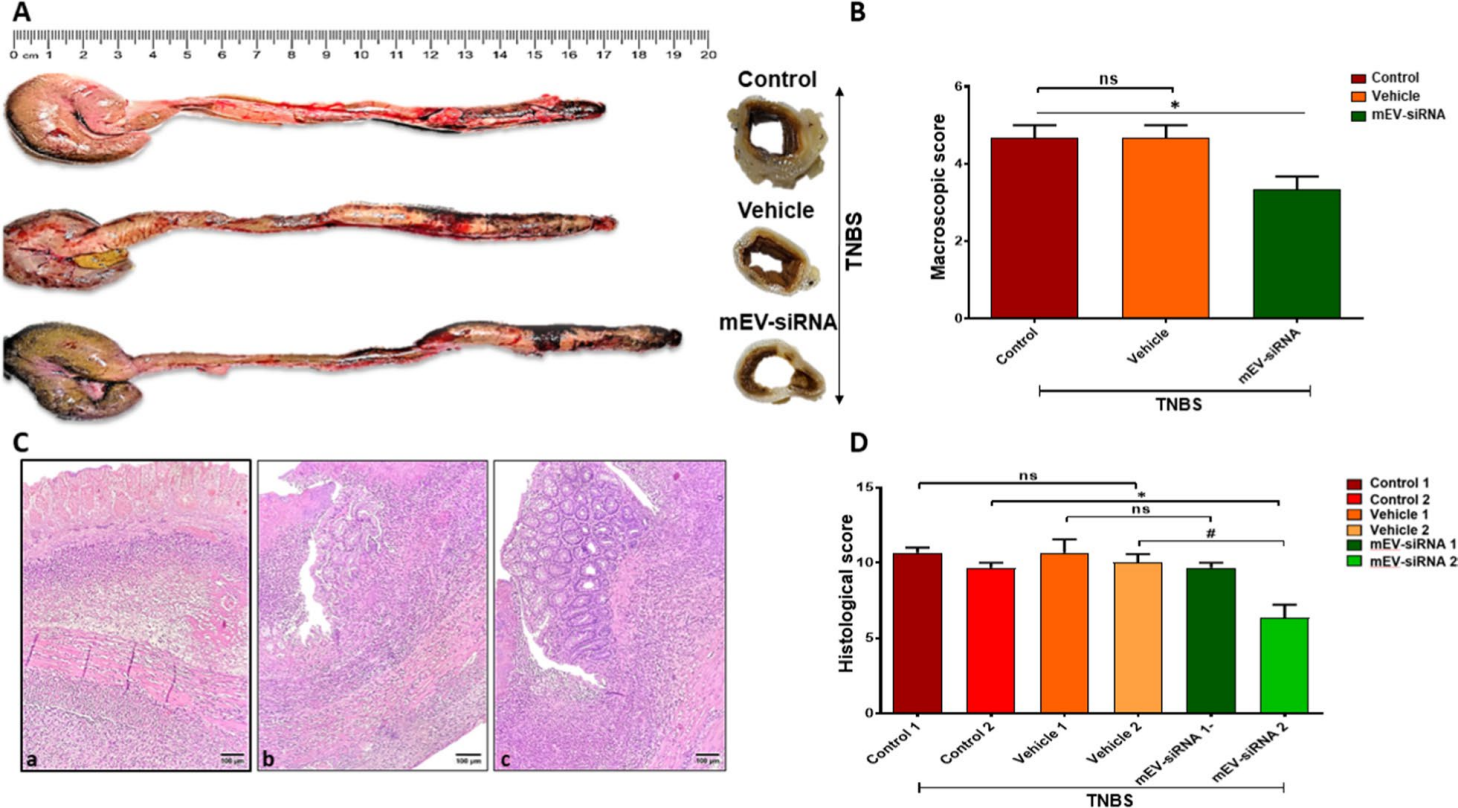
In vivo efficacy of milk extracellular vesicles (mEVs) loaded with anti-tumour necrosis factor alpha (TNFα) small interfering RNA (siRNA) in a rat model of ulcerative colitis. **A** Macroscopic presentation. **B** Macroscopic Score. **C** Histological sections (H&E 100x); **a** control, **b** vehicle (unloaded mEVs) and **c** anti TNFα siRNA loaded mEVs. **D** Histological Score. ‘Vesicle’ denotes treatment with electroporated, non-drug loaded mEVs at the same dose as siRNA loaded mEVs. Ulcerative colitis was induced *via* administration of 4,6-trinitrobenzene-sulfonic acid (TNBS). Data are presented as mean ± SD (n = 3). * Indicates *p* < 0.05

The effect of administration of anti-TNFα siRNA-loaded mEVs in a rat model of IBD, specifically TNBS-induced ulcerative colitis, is shown in Fig. 5. Treatment with anti-TNFα siRNA-loaded mEVs demonstrated an effect in the distal colon through the reduction of length of major site of inflammation, presented as macroscopic score (Fig. 5A, B and p < 0.05). Moreover, in the treated group a morphological improvement in histological sections was encountered, particularly when comparing the proximal sections of the distal colon between groups [Fig. 5D, ‘mEV-siRNA 2’ *versus* ‘Control 2’ or ‘Vehicle 2’ (mEVs alone, electroporated but non-drug loaded) vs., *p* < 0.05], which corresponds to the macroscopic findings. Foci of non-necrotic mucosa were present in the treated group, as shown in Fig. 5b, c, as opposed to non-treated TNBS induced colitis groups, which presented with diffuse mucosal and submucosal necrosis and severe inflammation and fibrosis (Fig. 5a) (5D, Control 1 or Vehicle 1 vs. mEV-siRNA 1). The significant morphological improvement in the proximal segment of the distal colon in the treated group suggests that mEVs successfully delivered functional anti-TNFα siRNA cargo, to which the effect is attributed. Our hypothesis is that anti-TNFα siRNA-loaded mEVs permeate the intestinal epithelium and influence cytokine production by immune cells, thereby alleviating inflammation and promoting barrier repair [30, 31]. Intestinal epithelial transport of siRNA-loaded mEVs was also confirmed in Caco-2 monolayers, whereby we tracked fluorescently labelled siRNA (instead of mEVs) (Additional file 1: Fig. S5), noting that mEV-loaded siRNA outperformed the transport of siRNA alone. It must be noted that in this proof-of-concept in vivo study, only a single dosage regimen of anti-TNFα siRNA-loaded mEVs was tested and future studies should optimise the treatment timeline, together with decreasing the TNBS dose used to induce inflammation so to obtain less necrosis, enabling better delineation of morphological findings following treatment. It is also critical to note that successful siRNA response (indicating successful delivery) was observed despite the low loading efficiency achieved with electroporation (~ 5%). There is therefore significant scope to further improve the therapeutic response to siRNA-loaded mEVs though dose optimisation and/or improvement of loading efficiency.

## Conclusions

Overall, our work confirms that mEVs are highly competent at transporting across the human intestinal epithelium and this property is not compromised by their treatment in intestinal fluids (hence indicating stability). Importantly, the work for the first time utilizes human-derived apical-out IEOs and IEO monolayers to demonstrate apical-to-basolateral transport of mEVs, which demonstrates the potential of mEVs as nanocarriers for intestinal epithelial delivery of biotherapeutic cargo, which would otherwise have poor delivery. The efficient induction of gene silencing in macrophages by siRNA loaded-mEVs, as well as a clearly efficient ability to permeate the intestinal epithelium indicates the therapeutic potential of mEVs. Using inflammatory bowel disease (IBD) as an example application, we show that administration of anti-TNFα siRNA-loaded mEVs reduced inflammation in a rat model of IBD. Therefore, mEVs could act as safe systems of natural origin that could enable oral delivery of nucleic acid therapies, such as RNA, or inform the design of synthetic delivery systems for such application.

## Methods

### Materials

Bovine pasteurized skimmed milk was purchased from a local grocery (Sainsbury’s). QuantiPro™ BCA Assay Kit, Triton X-100, Sodium Dodecyl Sulfate (SDS), *N,N*-Dimethylforamide Dulbecco’s Modified Eagle’s Medium (DMEM), Hank’s Balanced Salt Solution (HBSS), fetal bovine serum (FBS, non-USA origin), non-essential amino acids, antibiotic/antimycotic solution, N-Acetyl-L-cysteine, Nicotinamide, Gastrin I human, paraformaldehyde, Fluoroshield™ DAPI, low temperature gelling agarose, fluorescein isothiocyanate–dextran with molecular weight of 10k (FD10), MISSION® siRNA Fluorescent Universal Negative Control #1, Cyanine 5 and X-tremeGENE™ 360 Transfection Reagent were obtained from Merck (Dorset, UK). qEV original 35 nm size exclusion chromatography (SEC) was purchased from Izon Science (Lyon, France). Fasted- and Fed-State Simulated Intestinal Fluids (FaSSIF and FeSSIF, respectively) were purchased from Biorelevant (London, UK). TrypLE™ Express Enzyme, Advanced DMEM/F-12, HEPES (1 M), GlutaMAX™, Penicillin-Streptomycin, B-27™ Supplement (50X), Human Epidermal Growth Factor (EGF) Recombinant Protein, A83-01, Opti-MEM™ I Reduced Serum Medium, KDalert™ GAPDH Assay Kit, Silencer™ Select GAPDH Positive Control siRNA, Silencer™ Select Negative Control siRNA, ZO-1 polyclonal antibody and chicken anti-rabbit IgG (H + L) cross-adsorbed secondary antibody, Alexa Fluor™ 488 were bought from Thermo Fisher Scientific (Waltham, MA, USA). Goat Anti-Mouse IgG H&L (Alexa Fluor™ 594) was purchased from Abcam (Cambridge, UK). Tris-EDTA (TE) buffer was purchased from Promega (Southampton, UK). Caco-2 cells and macrophages (J774A.1) were purchased from European Collection of Cell Cultures (ECACC, Salisbury, UK). ExoGlow™-Protein EV Labeling Kit (Red), ExoQuick™ reagent and Exo-Check™ Exosome Antibody Array kit were purchased from System Biosciences (Palo Alto, CA, USA). 6.5 mm Transwell® with 0.4 μm pore polycarbonate membrane inserts and Corning® Matrigel® Growth Factor Reduced (GFR) Basement Membrane Matrix were purchased from Corning (Glendale, AZ, USA). Recombinant Human Noggin was purchased from PeproTech (London, UK). IntestiCult™ Organoid Growth Medium (Human), SB202190, and Y27632 was purchased from STEMCELL Technologies (Cambridge, UK). 24-well PET inserts with 0.4 μm pore size were purchased from SARSTEDT (Nümbrecht, Germany). Cultrex® Reduced Growth Factor Basement Membrane Extract Type 2, PathClear® (BME2) and DAPT were purchased from Bio-Techne Ltd. (Oxford, UK).

### mEV isolation, characterization and labelling

mEVs were isolated from skimmed bovine milk by differential ultracentrifugation process according to the methods described by previous studies [32, 33]. Briefly, 70 mL of milk was centrifuged at 13,000 × g for 30 min at 4 ºC with Optima XPN-80 Ultracentrifuge (Beckman Coulter, Type 45 Ti fixed angle rotor) to remove fats and casein. The whey was then centrifuged at 100,000 × g for 60 min to pellet large particles. The supernatant was filtered by 0.2 μm filter to remove large particles and further centrifuged at 135,000 × g for 90 min, producing mEVs pellets which were washed with PBS once and resuspended in 1 mL sterile PBS. Resuspended mEVs were then purified by SEC and resulting 500 µL of fractions containing mEVs were collected for downstream application. Purified mEVs in sterile PBS could be stored at − 80 ºC for up to 3 months. Total protein concentration of mEV samples was determined by QuantiPro™ BCA Assay Kit following the manufacturer’s instructions. Size and particle concentration (yield) were determined by NTA (Malvern Nanosight LM-10, UK). Surface charge (Zeta-potential) was measured by Malvern Zetasizer (Malvern, UK). The expression of exosome protein markers was determined using Exo-Check™ Exosome Antibody Array Kit following manufacturer’s instructions.

The morphological features of mEVs were examined by TEM. Briefly, 300-mesh carbon-coated copper grids were pre-treated by glow discharge (negative charge). 3 µl of mEVs were applied onto the grids and incubated at room temperature for 1 min, after which were stained with 3% uranyl formate for additional 1 min. Images were acquired on JEM-1400 flash (JEOL, Japan) at an acceleration voltage of 80 kV.

To image and quantify the transport of mEVs across intestinal epithelial models, mEVs were labelled using an ExoGlow™-protein EV labeling kit (Red). Briefly, 1 µL of the labelling dye was added to 500 µL mEVs suspension (0.4–1.0 mg/mL) and incubated at 37 °C for 20 min with shaking. Thereafter, 167 µL ExoQuick™ reagent was added to the mixture and incubated at 4 °C overnight to precipitate mEVs. Thereafter, the mixture was centrifuged at 10,000 × rpm for 10 min to remove excess labelling dye and pellet the labelled mEVs which were then resuspended in sterile PBS.

### Preparation of liposomes

Liposomes were prepared using the following lipids: 1,2-dipalmitoyl-*sn*-glycero-3-phosphocholine (DPPC); 1,2-dioleoyl-*sn*-glycero-3-phosphoethanolamine (DOPE); 1,2-distearoyl-*sn*-glycero-3-phosphoethanolamine-*N*-(7-nitro-2-1,3-benzoxadiazol-4-yl) (ammonium salt) (NBD-DSPE); and Rhodamine PE (Rho-PE), with the molar ratio of 67%:30%:1.5%:1.5%. All lipids were dissolved in chloroform and were added to a round bottom flask. The organic solvent was evaporated by a rotary evaporator to create the lipid film which was dried under vacuum overnight. Thereafter, lipid film was hydrated by HEPES buffer (4 mM, pH 7.4) with a final lipid concentration of 1 mg/mL and 10 freeze-thaw cycles (freezing in liquid nitrogen and thawing by sonication at 40 ℃ for ∼5 min) applied to develop and homogenize the liposomes.

### Comparison of transport of mEVs and liposomes in intestinal Caco-2 monolayers

The purpose of these studies was to compare intestinal epithelial transport of mEVs versus a synthetic lipid nanoparticle system, namely liposomes of similar size (~ 100 nm). A comparison was also made with the free fluorescent dye which was used to label mEVs. Epithelial transport was determined in differentiated intestinal Caco-2 monolayers as a commonly used intestinal model. Caco-2 cells were cultured on Transwell inserts for 21 days, with measurement of TEER by EVOM (World Precision Instruments, Sarasota, FL, USA) to ensure monolayer barrier integrity. Prior to the transport study, the culture medium was replaced with HBSS and cells incubated for 45 min at 37 °C and 5% CO_2_ atmosphere for equilibration. Thereafter, 500 µL of labelled mEVs at 0.05 mg/mL protein concentration, liposomes at 0.05 mg/mL lipid concentration, and fluorescent dye the concentration of which was adjusted to produce same fluorescent signal as that of mEVs, were added to the apical side of monolayers for 120 min. During the incubation, 100 µL basolateral solution was sampled regularly (at 30 min intervals), with the sampled solution replaced with HBSS. mEVs and labelling dye in the sampled basolateral solution were quantified by fluorescence using plate reader (excitation 565 nm; emission 615 nm), and liposomes were quantified by Rhodamine fluorescence with excitation wavelength of 530 nm and emission wavelength of 588 nm.

### Effect of simulated intestinal fluids (SIFs) on mEVs physicochemical characteristics and transport across Caco-2 monolayers

FaSSIF (pH 6.5) and FeSSIF (pH 5.0) were used as simple, commercially available models of small intestinal fluids. SIFs were prepared according to the manufacturer’s instructions. 100 µL of mEV suspension at 1 mg/mL protein concentration was incubated in 400 µL of SSIFs at 37 ºC with gentle shaking for 1.5 h. After digestion, mEVs were recovered *via* centrifugal ultrafiltration with 100 kDa Amicon Ultra-0.5 Centrifugal Filter Unit (Merck, Dorset, UK) by four sequential centrifugations for 10 min each at 10,000 × rpm. 500 µL of PBS was added between each spin to wash off the remaining debris from the digestion solutions. Post treatment with SSIFs, mEVs were resuspended in 200 µL PBS and characterized for size and Zeta-potential.

Harvested mEVs were then labelled with a fluorescence dye to enable quantitation of transport. Labelling was achieved using an ExoGlow^TM^-protein EV labeling kit (Red) as mentioned above. Thereafter, 500 µL of labelled mEVs at 0.05 mg/mL protein concentration that were previously treated with SIFs or PBS (control) were added to the apical side of monolayers for 90 min. Transport was determined using the same process as mentioned above.

### Culture of 3D apical-out intestinal epithelial organoids (IEOs)

IEOs were a kind gift from Dr Vivian Li (Francis Crick Institute, London, UK). They were generated by Dr Li from tissues collected from a 2-year-old female patient with ethical approval (Research Ethics Committee reference 04/Q0508/79). IEOs were defrosted from liquid nitrogen stock in 37 °C and then centrifuged at 800 × g for 5 min immediately. IEOs pellets were resuspended in Matrigel and seeded on pre-warmed 24 wells plate with 50 µL droplet per well and supplemented with 0.5 mL IEOs growth medium: Advanced DMEM/F-12 with 10 mM HEPES, 1X GlutaMAX™, 50% WNT3A conditioned medium (in house production), 20% R-Spondin-1 conditioned medium (in house production), 1.25 mM N-Acetyl-L-cysteine, 10 mM Nicotinamide, 1X B-27™ Supplement, 150 ng/mL Recombinant Human Noggin, 50 ng/mL Human EGF Recombinant Protein, 10 nM Gastrin I human, 0.5 µM A83-01 and 10 µM SB202190. The growth medium was changed every 2–3 days. IEOs cultures were passaged every 7–10 days, and 10 µM Y-27,632 was added to the growth medium for the first 2–3 days after passage.

Apical-out 3D IEOs were generated by non-Matrigel culturing. PET inserts with 0.4 μm pore size were used for this purpose. Briefly, IEOs in Matrigel were broken up by pipette tip gently and centrifuged at 800 × g for 5 min to remove the medium and most of Matrigel. IEOs pellets were resuspended in TrypLE™ with 500 µL/well and incubated in 37 °C for 10 min to disassociate IEOs into single cells. Thereafter, IEOs were centrifuged again to remove TrypLE™ and the pellets were resuspended in IEOs growth medium. 70 μm cells strainer was used to filter the suspension and single IEOs cells were collected. To develop apical-out IEOs, dissociated cells were seeded on 24-well PET inserts with 1.0 × 10^5^ – 5.0 × 10^5^ cells/well (100 µL/well) and cultured for 10 days. The growth medium was changed every 2–3 days, and 10 µM Y-27,632 was added for the first 2–3 days after seeding. To create conventional, basolateral-out IEOs on the same inserts, 25 µL/well Matrigel was used to coat the surface of inserts. After incubated the coated-inserts in 37 °C for 15 min, IEOs single cells were seeded and cultured on inserts with same procedures as apical-out IEOs.

### Culture of intestinal epithelial organoid (IEO) monolayers

IEOs were generated from mucosal biopsies (i.e. duodenum, terminal ileum, sigmoid colon) obtained from a patient with mild chronic gastritis. Ethical approval for the study was obtained (Research Ethics Committee reference 17/EE/0265). IEOs (‘IBD-IEOs’) were cultured as previously reported [34], with minor modification. Briefly, IEOs were defrosted from liquid nitrogen stock at 37 °C, then immediately added to warm AF+++ medium (Advanced DMEM/F-12 with 10 mM HEPES, 1X GlutaMAX™, and 1% Penicillin-Streptomycin (v/v)). After centrifugation at 800 × g for 5 min, IEO pellets were resuspended in Matrigel and seeded on pre-warmed 48 wells plate (3548, Corning) with 20 µL droplet per well and supplemented with 0.25 mL IntestiCult™ Organoid Growth Medium (Human) (growth medium). The growth medium was changed every 2–3 days. IEOs cultures were passaged every 7–10 days, and 10 µM Y-27,632 was added to the growth medium for the first 2–3 days after passage. Following culture for 4–5 days in growth medium, IEOs were differentiated by differentiation medium for another 3–4 days. Differentiation media was prepared by AF+++ medium supplemented with 50 ng/mL Human EGF Recombinant Protein, 100 ng/mL Recombinant Human Noggin, 10 nM Gastrin I human, 500 nM A83-01, 10 µM Y27632, 5 µM DAPT, 1 mM N-Acetyl-L-cysteine and 1X B27 [22]. The differentiation medium was changed every 2 days.

IEO monolayers were generated as previously reported, with some modifications [35]. Firstly, the 6.5 mm transwells inserts (PET for imaging and polycarbonate for transport study) were coated with 40X diluted BME2. Specifically, BME2 was diluted with cold AF+++ medium by 40X and 150 µL of diluted BME2 was added to each well followed with incubation in 37 °C for 1–2 h. Diluted BME2 medium mixture was removed from wells before adding the cells. Organoids which were not excessively large were used to generate monolayer model. Briefly, IEOs in Matrigel were broken up and resuspended in cold AF+++ medium. After centrifugation (800 x g, 5 min), organoid pellets were resuspended in TrypLE™ with 250 µL/well and incubated in 37 °C for 10 min to disassociate them into single cells. The suspension was pipetted during the incubation to help with the disassociation of organoids. Thereafter, cold AF+++ medium was added to the TrypLE™ mixture and centrifuged at 800 × g for 5 min. IEO cell pellets were resuspended in cold AF+++ medium and filtered with 70 μm cells strainer. After centrifugation, cells were resuspended in growth medium. To develop IEO monolayers, 0.8–1.5 × 10^6^ cells/mL were added to the apical side of transwell inserts (150 µL/well) and 600–800 µL growth medium (depending on plate types) was added basolaterally. Growth medium was changed every 2–3 days, and 10 µM Y-27,632 was added for the first 2–3 days after seeding. Differentiation of IEO monolayers was induced as mentioned above. Cell monolayer growth was monitored by measuring TEER.

### Confocal immunofluorescence imaging of apical-out IEOs and IEO monolayer models

IEOs cultured in Matrigel, 3D apical-out IEOs and IEO monolayers were washed with 0.01 M PBS for 3 time and fixed with 4% paraformaldehyde for 20 min at room temperature followed permeabilized with 0.1% Triton-X 100 for 5 min. Thereafter, IEOs were blocked with blocking buffer (5% (w/v) skimmed dry milk powder with 0.5% Triton-X 100 in 0.01 M PBS) for 1 h. IEOs were then incubated with primary antibodies (1:150 ZO-1 polyclonal antibody and 1:150 MUC2 monoclonal antibody diluted in 1% (w/v) skimmed dry milk powder with 0.5% Triton-X 100) at 4 °C overnight and then incubated with secondary antibodies (1:500 goat anti-rabbit Alexa Fluor™ 488 lgG and 1:500 goat anti-mouse Alexa Fluor™ 594 IgG diluted in 1% (w/v) skimmed dry milk powder with 0.05% Triton-X 100) for 1 h at room temperature. Finally, IEOs were treated with Fluoreshield™ DAPI to stain nuclei. To enable imaging, transwell membrane-supported IEOs were cut off and placed on a 24-well plate with polymer coverslip bottom (µ-Plate 24 Well Black ID 14 mm, Ibidi, Gräfelfing, Germany). Then, 0.5% (w/v) low temperature gelling agarose at 30 °C was added drop-wise to cover and immobilize the membrane. IEOs cultured in Matrigel were imaged directly after immunostaining. Images were collected by the 20 X or 40 X water objective on Opera Phenix™ High Content Screening System (PerkinElmer, Waltham, MA, US).

### mEV transport across apical-out IEOs and IEO monolayers

To evaluate the transport of mEVs through 3D apical-out IEOs, labelled mEVs were diluted in IEOs growth medium to 0.05 mg/mL and applied to 3D apical-out IEOs for 4 h at 37 °C and 5% CO_2_ atmosphere. Thereafter, the growth medium containing mEVs was removed and IEOs immunostained and imaged as described above.

Prior to the study of mEVs transport across IEOs monolayers, the integrity of monolayers was evaluated by determining FD10 permeability. To do this, culture medium was replaced by HBSS and monolayers incubated for 45 min at 37 °C and 5% CO_2_ atmosphere for equilibration. Thereafter, 150 µL of 1 mg/mL FD10 in HBSS was added to the apical side of monolayers for 160 min. During the incubation, 100 µL basolateral solution was sampled regularly (at 40 min intervals), with the sampled solution replaced with HBSS. FD10 was quantified by fluorescence at 490 nm/520 nm excitation/emission wavelengths. The transport of labelled mEVs through IEO monolayers was determined in a similar manner, with mEVs quantitation using fluorescence at excitation wavelength of 565 nm and emission wavelength of 615 nm. For confocal imaging of the IEO monolayers after mEV transport, cells on Transwell filters were processed for immunostaining and confocal imaging as mentioned above.

### siRNA Loading of mEVs

siRNA was loaded into mEVs by electroporation, which was optimized based on a previous report [36]. Specifically, 150 µg mEVs were mixed with 0.58 nmol siRNA, followed by the addition of the electroporation buffer (1:1 v/v ratio to mEVs-siRNA mixture). The electroporation cuvette was incubated in ice for 10 min. Electroporation was performed at 400 V/200 µF on a Gene pulser System (Bio-Rad Laboratories, Watford, UK). Unloaded siRNA was removed *via* ExoQuick™ reagent or ultracentrifugation (135,000 × g for 90 min). Cyanine 5 fluorescent siRNA was used to calculate the loading efficiency by Eq. (1):

1 $$\% \,~loading~efficiency = 100~ \times ~\frac{{amount~of~loaded~siRNA}}{{amount~of~added~siRNA}}$$

### siRNA transfection

J774A.1 macrophage cells were seeded on 96-wells plate with 5000 cells/well and cultured for 24 h to ~ 50% confluence. GAPDH siRNA loaded-mEVs were diluted to 0.05 and 0.02 mg/mL (corresponding to the siRNA concentration of 0.010 and 0.004 nmol/mL calculated by the loading efficiency) with Opti-MEM™ I Reduced Serum Medium and incubated with cells for 48 h at 37 °C and 5% CO_2_ atmosphere. Thereafter, GAPDH activity was measured by KDalert™ GAPDH Assay Kit according to the manufacturer’s instructions. Negative siRNA loaded-mEVs were applied as negative control group, and GAPDH siRNA transfected with commercial transfection reagent (X-tremeGENE™ 360 Transfection Reagent, 2.5 µl/mL) was applied as positive control group. The % remaining GAPDH gene expression was calculated with Eq. (2):

2 $$\% ~\,remaining~expression{\text{~}} = 100{\text{~}} \times {\text{~}}\frac{{\Delta \,fluorescence~of~GAPDH}}{{\Delta \,fluorescence~of~Negative}}$$

Where *∆fluorescence of GAPDH* and *∆fluorescence of Negative* are fluorescence increases within 4 min for samples and negative control group, respectively.

### In vivo evaluation of siRNA-loaded mEVs

Animal studies were carried out under the University guidelines for the care of experimental animals and were approved by Research and Ethics Committee of the Faculty of Medicine at the University of Prishtina, Kosovo. Male Wistar rats weighing 380–420 g were used in this study. Rats were housed per each group in a Digital Ventilated Cages (DVC®, Tecniplast, Italy) under 20–22 °C and had a 12:12-h photoperiod with an access to standard laboratory chow and water ad libitum. The induction of colitis model was performed by 4,6-trinitrobenzene-sulfonic acid (TNBS, Sigma-Aldrich) based on a previous study [37]. To this end, TNBS was dissolved in 50% ethanol to a concentration 30 mg/ml. Rats were fasted 24 h and 40 mg/kg of TNBS were administered intrarectally 8 cm proximal to the anus (total volume of 500–560 µL) by a lubricated silicone catheter. For TNBS administration, rats were anesthetized with Ketamine-xylazine cocktail (day 0). Animals were maintained in a head side down situation for 1 min to prevent any leakage of solution. Rats were treated with anti-TNFα siRNA-loaded mEVs (‘mEV-siRNA’), unloaded mEVs, which were electroporated using the same conditions as siRNA loaded mEVs (‘vehicle’), or PBS (‘control’), starting 24 h after TNBS administration for four days (single oral gavage dosage of 200 µL, day 1–4). siRNA loaded and unloaded mEVs were administered in PBS and the same volumes were administered in all groups. On day 6 of experiment rats were euthanized with asphyxiation in CO_2_ and the colon was dissected carefully for further macroscopic and microscopic evaluation.

The severity of macroscopic changes was evaluated by an independent observer who was blinded to the treatment. For each animal, the distal 10 cm portion of the colon was cut perpendiculary and slightly cleaned in PBS to remove faecal residues. Scores were assigned based on clinical features of the colon as per previous recommendations adapted to our findings (score 0–5: 0 (no damage), 1 (hyperaemia without ulceration), 2 (hyperemia with ulceration up to ˂ 4 cm along the colon), 3 (major sites of inflammation ≥ 4 cm to ˂ 5 cm along the colon), 4 (major sites of inflammation ≥ 5 cm to ˂ 6 cm, 5 (major sites of inflammation ≥ 6 cm) [38]. For histological changes, two perpendicular sections were taken from the distal 10 cm portion of the distal colon of each animal. One section from the zone with the most extensive macroscopic changes and one section from the most proximal zone of macroscopic changes in the distal colon. Tissue samples were fixed in 4% buffered paraformaldehyde, dehydrated in grade ethanol, embedded in paraffin and cut into 5 mm sections using a rotary microtome. Thereafter, sections were mounted on clean glass slides and stained with haematoxylin and eosin (H&E). All tissue sections were examined with light microscopy for characterization of histopathological features by an experienced pathologist. The histopathological features were semiquantitatively scored as per previous recommendations adapted to our findings (score 0–12, 0- (normal), 1–4 (light microscopic changes), 5–9 (moderate histological changes), 10–12 (extensive histological changes)) for the presence of transmucosal and submucosal necrosis and ulceration extending through the muscularis mucosae (Grade: 0—no presence; 1—focal necrosis or ulceration; 2—diffuse necrosis with foci of non-necrotic mucosa with or without ulceration; 3—diffuse necrosis with or without ulceration); oedema and transmural inflammatory cells such as polymorphonuclear leukocytes, lymphocytes, and eosinophils; muscularis externa thickness and serosal fibrosis (Grade: 0—normal, 1—light, 2—moderate, 3—extensive) [38, 39].

### Statistical analysis

Data were analyzed using GraphPad Prism®. Data is presented using the mean ± standard deviation (SD) using at least three technical replicates and repeat experiments. Statistical analysis was performed by unpaired Student’s t-test or ANOVA, and normality distribution of data was checked before analysis. Differences with a *p*-value lower than 0.05 were taken as significant. The * and ** nomenclature were used to indicate *p* < 0.05 and *p* < 0.01, respectively.

### Supplementary Information

**Additional file 1:**  Characterisation of bovine milk extracellular vesicles (mEVs). Transport of mEVs, liposomes and free fluorescent dye across Caco-2 monolayers, brightfield image and confocal image of human (biopsy-derived) intestinal epithelial organoids (IEOs) cultured in Matrigel extracellular matrix, brightfield image of human terminal ileum IEOs as 2D monolayers on Transwell inserts, and transport of fluorescent Cy5 siRNA electroporated-mEVs and siRNA alone across Caco-2 monolayers) is available in the online version of this article.

## Data Availability

All data needed to evaluate the conclusions in the paper are present in the paper and/or the Additional files.
