# Supplementary material for: Probing milk extracellular vesicles for intestinal delivery of RNA therapies
Source: J Nanobiotechnology. 2023 Nov 3;21:406. doi: 10.1186/s12951-023-02173-x (PMC10623793; doi:10.1186/s12951-023-02173-x)
Supplement: Supplementary file 1 — Additional file 1: Characterisation of bovine milk extracellular vesicles (mEVs). Transport of mEVs, liposomes and free fluorescent dye across Caco-2 monolayers, brightfield image and confocal image of human (biopsy-derived) intestinal epithelial organoids (IEOs) cultured in Matrigel extracellular matrix, brightfield image of human terminal ileum IEOs as 2D monolayers on Transwell inserts, and transport of fluorescent Cy5 siRNA electroporated-mEVs and siRNA alone across Caco-2 monolayers) is available in the online version of this article. [file 12951_2023_2173_MOESM1_ESM.docx]

Supporting Information

**Probing Milk Extracellular Vesicles for Intestinal Delivery of RNA Therapies**

*Yunyue Zhang^1^, Mona Belaid^1^, Xiang Luo^1^, Armond Daci^2^, Rinë Limani^2^*, *Julia Mantaj^1,3^, Matthias Zilbauer^4,5^, Komal Nayak^4,5^, Driton Vllasaliu^1^**

1. Institute of Pharmaceutical Science, School of Cancer and Pharmaceutical Science, King’s College London, London SE1 9NH, United Kingdom.

2. Faculty of Medicine, University of Prishtina “Hasan Prishtina”, Prishtina 10000, Kosovo.

3. Present address: School of Life Sciences, Faculty of Science and Engineering, Anglia Ruskin University, Cambridge CB1 1PT, United Kingdom

4. Wellcome-MRC Cambridge Stem Cell Institute, University of Cambridge, Cambridge CB2 0AW, United Kingdom

5. Department of Paediatrics, University of Cambridge, Cambridge CB2 0QQ, United Kingdom

**
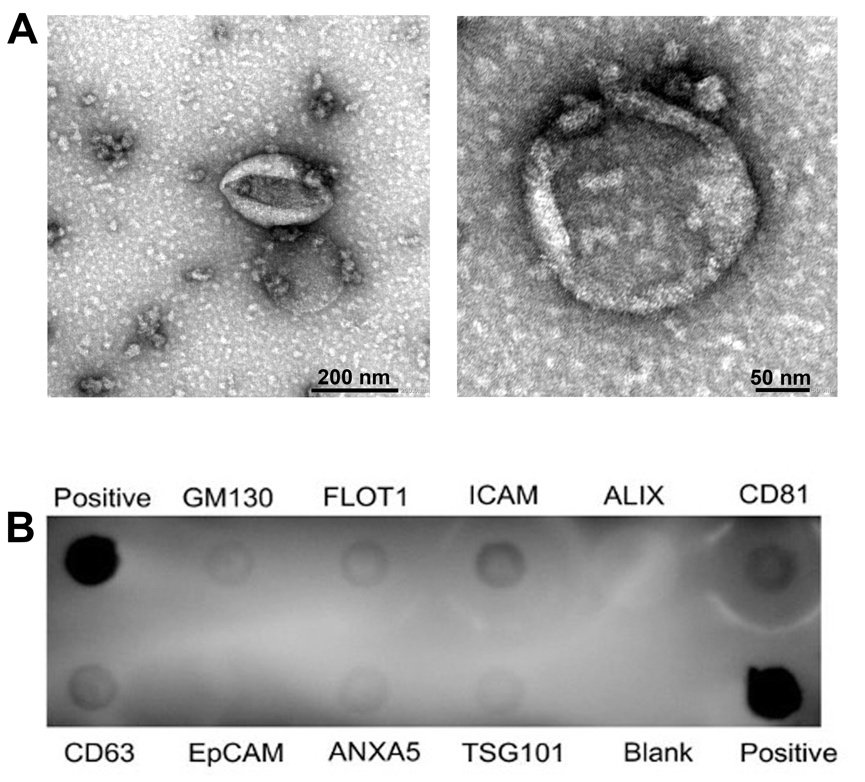
**

**Figure S1.** Characterisation of bovine milk extracellular vesicles (mEVs). (**A**) Morphological characterisation of mEVs by transmission electronic microscopy (TEM). (**B**) Expression of standard protein markers in mEVs, as determined by the Exo-Check™ Array. Protein marker tested: FLOT1 (Flotillin 1), ICAM (Intercellular Adhesion Molecule-1), CD81, CD63, ANXA5 (Annexin A5) and TSG101 (Tumour Susceptibility Gene 101). ‘Positive’ denotes labelled positive control for horseradish peroxidase detection which indicated that the detection reagents were working properly, and the blank spot serves as a background control.

**Figure S2**. Transport of bovine milk extracellular vesicles (mEVs), liposomes and labelling dye alone (for mEVs) across differentiated intestinal epithelial Caco-2 monolayers. Data shown as the mean ± SD, n=3.

**IEO Culture and Differentiation**

IEOs were generated from intestinal crypts isolated from mucosal biopsies of a patient with mild chronic gastritis and were cultured in Matrigel covered with IEOs growth medium. IEOs from different intestinal segments demonstrated different morphologies. **Figure S3A** and **S3D** show representative brightfield images of duodenum (‘Duo’) and terminal ileum (‘TI’) IEOs, respectively. Duodenal IEOs showed a cystic morphology with bright lumen, while terminal ileum IEOs produced more buds with an irregular shape. Immunofluorescence images (confocal microscopy) of undifferentiated Duo IEOs are shown in **Figure S3B**, whereas **Figure S3C** shows Duo IEOs following differentiation. The fluorescence signal associated with immunostaining of apical zonula occludens (ZO-1) tight junction proteins appears to be similar in both differentiated and undifferentiated Duo IEOs, while that of MUC2 mucin, which is secreted by goblet cells, was increased following differentiation. **Figure S3D and S3E** show undifferentiated TI IEOs, with the latter depicting immunofluorescence of ZO-1 and MUC2. The immunofluorescence-based determination of expression of these proteins is also shown in **Figure S3F** for differentiated TI IEOs. Similarly to Duo IEOs, the figure clearly indicates that differentiation of TI IEOs promotes MUC2 expression, as confirmed by a visibly increased fluorescence signal.


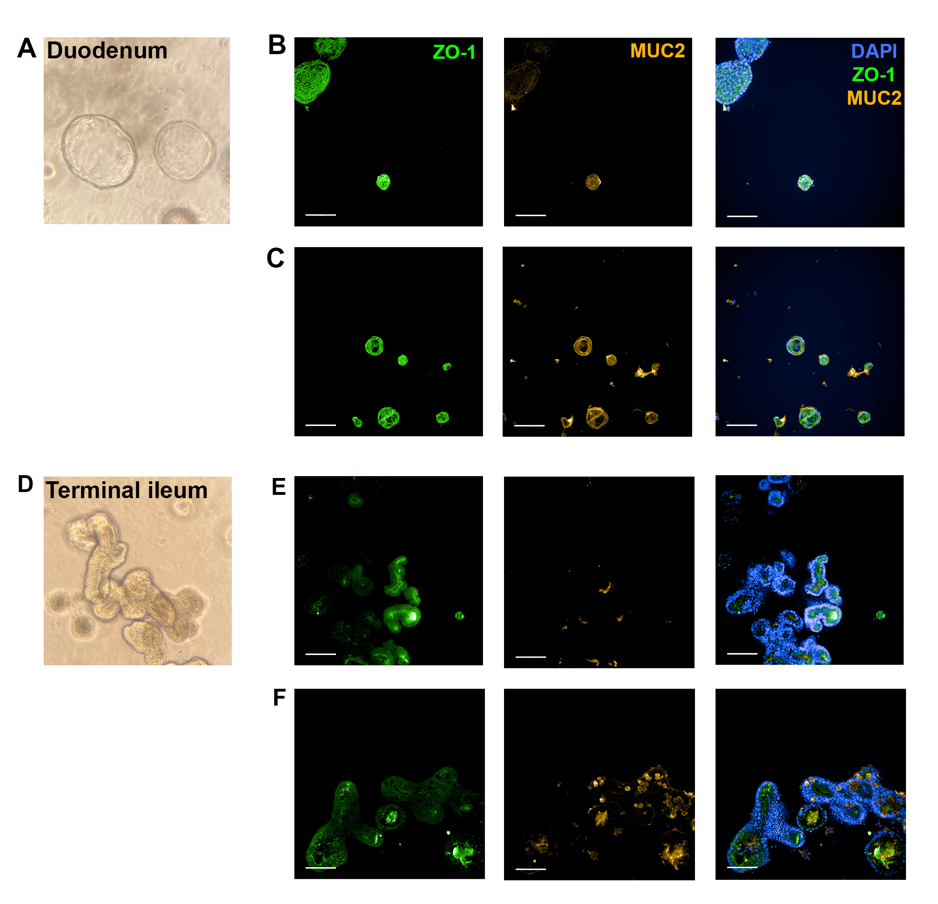


**Figure S3.** Human (biopsy-derived) intestinal epithelial organoids (IEOs) cultured in Matrigel extracellular matrix. **(A)** Brightfield image of duodenum IEOs. **(B)** Confocal immunofluorescent staining images of undifferentiated duodenum IEOs for apical zonula occludens (ZO-1) tight junction protein (green), MUC2 mucin (orange) and cell nucleus (DAPI, blue). **(C)** Confocal immunofluorescent staining images of differentiated duodenum IEOs. **(D)** Brightfield image of terminal ileum IEOs. **(E)** Confocal immunofluorescent staining images of undifferentiated terminal ileum IEOs. **(F)** Confocal immunofluorescent staining images of differentiated terminal ileum IEOs. Scale bars: 100 μm.


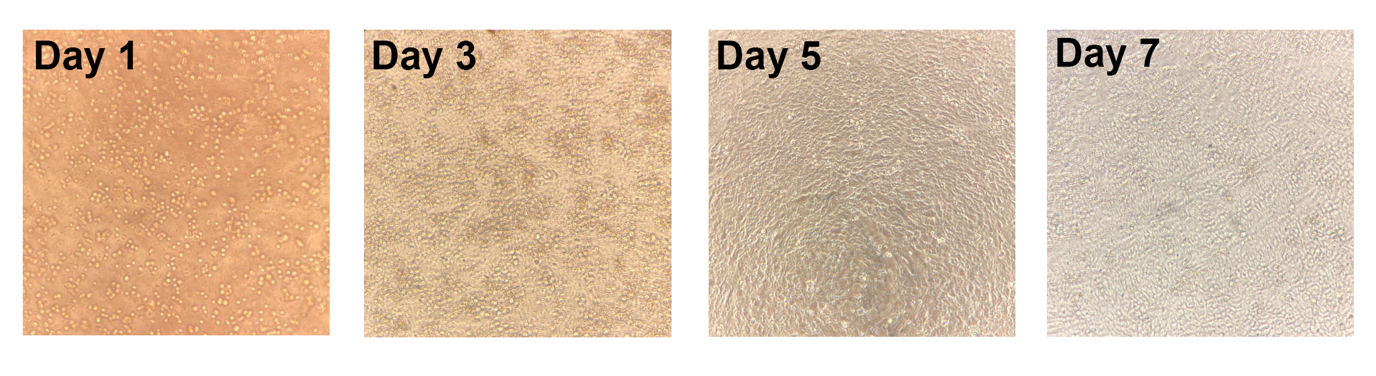


**Figure S4**. Brightfield images of human terminal ileum epithelial organoids (IEOs) as 2D monolayers on Transwell inserts of 1 day, 3 days, 5 days, and 7 days culturing after seeding (differentiated at day 5).

**Figure S5**. Transport of fluorescent Cy5 siRNA electroporated-bovine milk extracellular vesicles (mEVs), and siRNA alone across Caco-2 monolayers (intestinal epithelium). Transport percentage was calculated by the fluorescent signal of siRNA. Data shown as the mean ± SD (n=3). * indicates *p* < 0.05.
